# Supplementary material for: Identification of peptides from honeybee gut symbionts as potential antimicrobial agents against Melissococcus plutonius
Source: Nat Commun. 2023 Nov 24;14:7650. doi: 10.1038/s41467-023-43352-6 (PMC10673953; doi:10.1038/s41467-023-43352-6)
Supplement: Supplementary file 1 — Supplementary Information [file 41467_2023_43352_MOESM1_ESM.pdf]

Supplementary Information

Identification of peptides from honeybee gut symbionts as potential antimicrobial agents against *Melissococcus plutonius*

Haoyu Lang[1], Yuwen Liu[1], Huijuan Duan[1], Wenhao Zhang[1], Xiaosong Hu[1], Hao Zheng[1,*]

[1]College of Food Science and Nutritional Engineering, China Agricultural University, 100083 Beijing, China

*Address correspondence to Hao Zheng, hao.zheng@cau.edu.cn

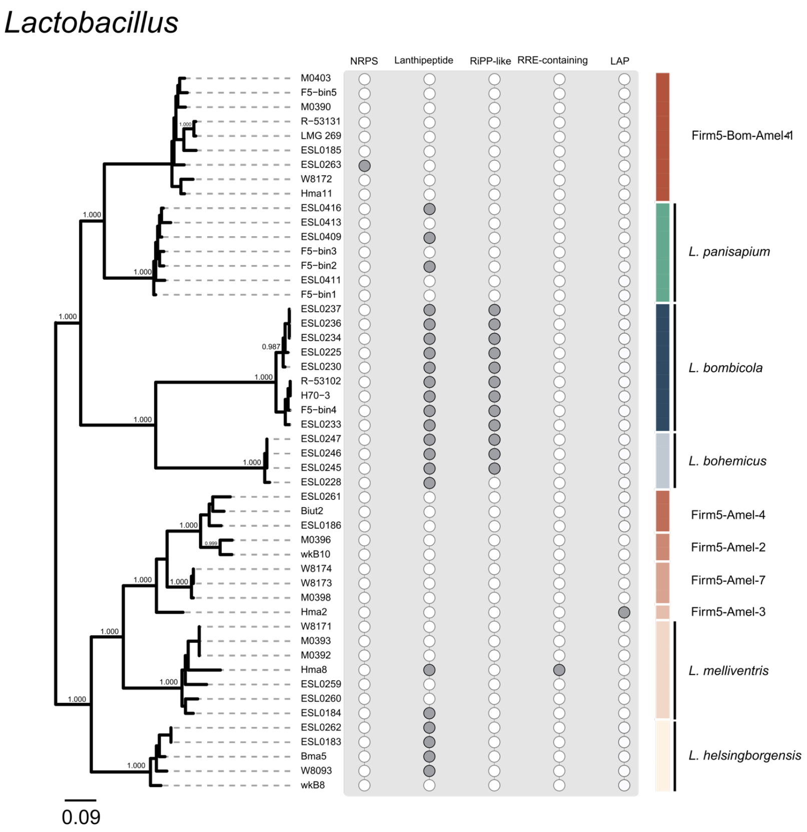

Supplementary Fig. 1. Genome phylogenies and species cluster classification of the *Lactobacillus* genus from honey and bumble bee guts. Cladograms on the left of each panel are maximum-likelihood trees inferred by GTDB-tk based on the amino acid sequences of bacterial marker genes. The presence of BGCs is indicated by grey circles.

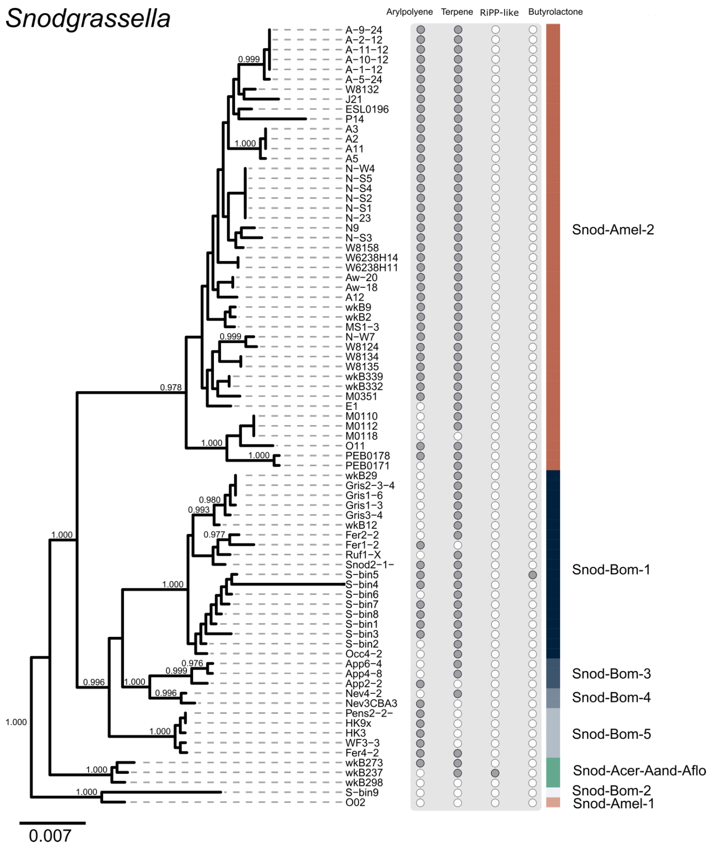

Supplementary Fig. 2. Genome phylogenies and species cluster classification of the *Snodgrassella* genus from honey and bumble bee guts. Cladograms on the left of each panel are maximum-likelihood trees inferred by GTDB-tk based on the amino acid sequences of bacterial marker genes. The presence of BGCs is indicated by grey circles.

Bifidobacterium

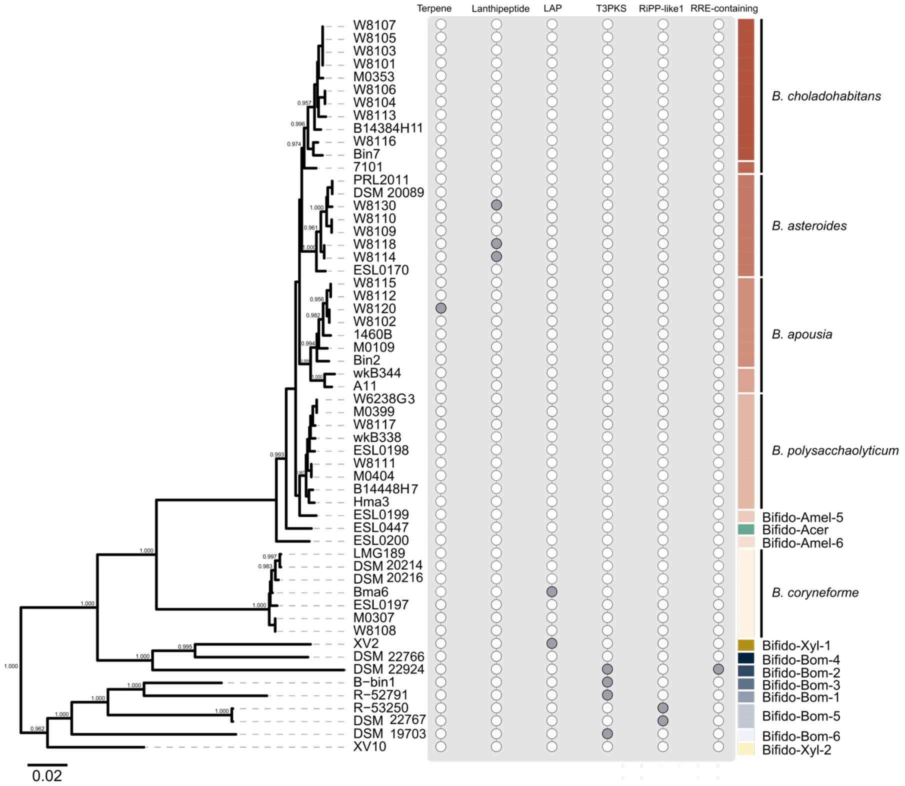

Supplementary Fig. 3. Genome phylogenies and species cluster classification of the *Bifidobacterium* genus from honey and bumble bee guts. Cladograms on the left of each panel are maximum-likelihood trees inferred by GTDB-tk based on the amino acid sequences of bacterial marker genes. The presence of BGCs is indicated by grey circles.

Bombilactobacillus

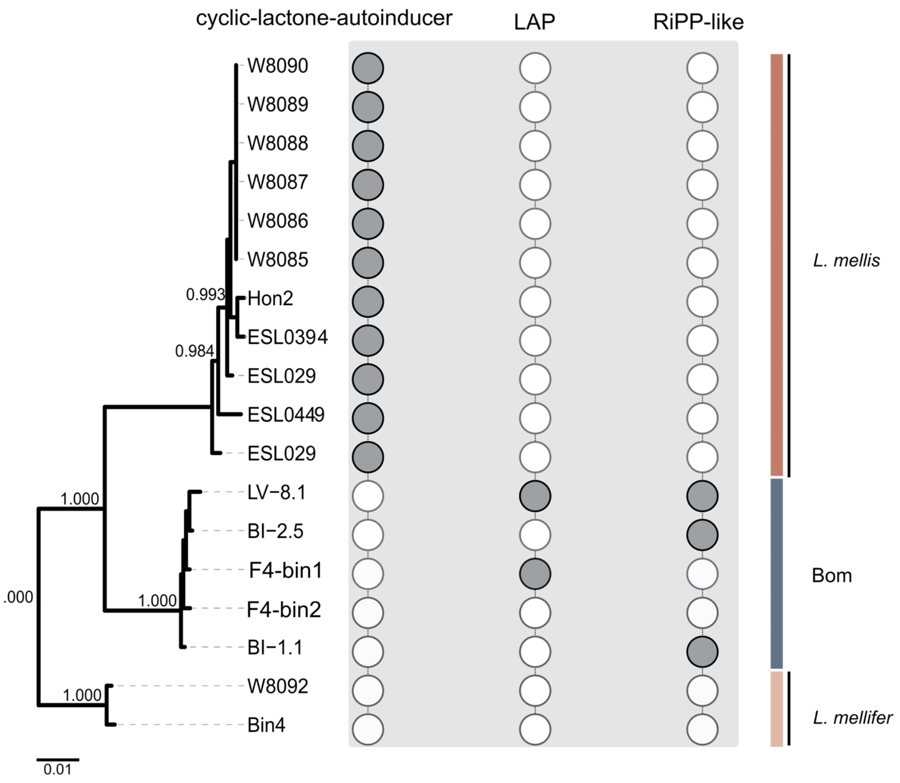

Supplementary Fig. 4. Genome phylogenies and species cluster classification of the *Bombilactobacillus* genus from honey and bumble bee guts. Cladograms on the left of each panel are maximum-likelihood trees inferred by GTDB-tk based on the amino acid sequences of bacterial marker genes. The presence of BGCs is indicated by grey circles.

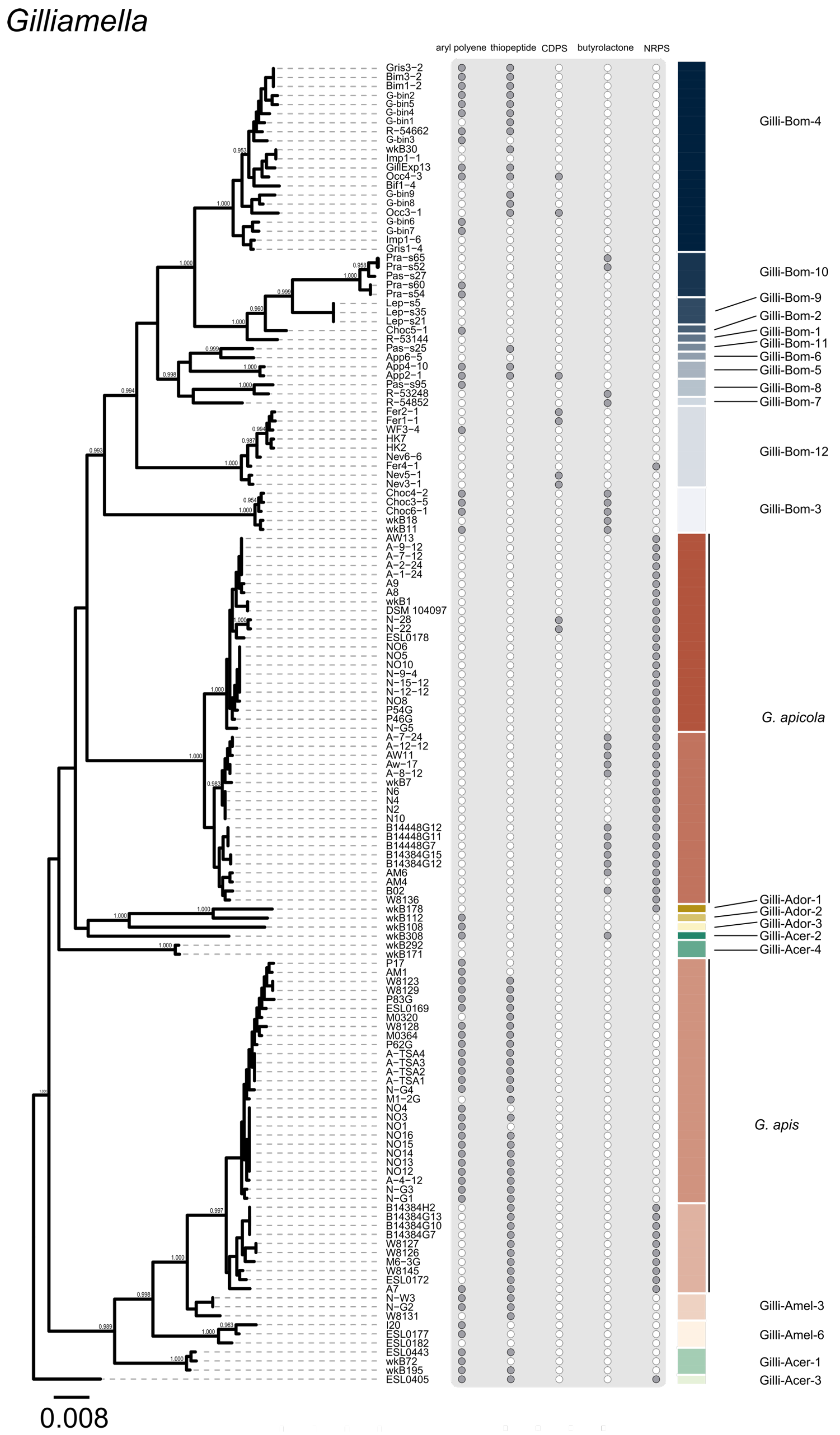

Supplementary Fig. 5. Genome phylogenies and species cluster classification of the *Gilliamella* genus from honey and bumble bee guts. Cladograms on the left of each panel are maximum-likelihood trees inferred by GTDB-tk based on the amino acid sequences of bacterial marker genes. The presence of BGCs is indicated by grey circles.

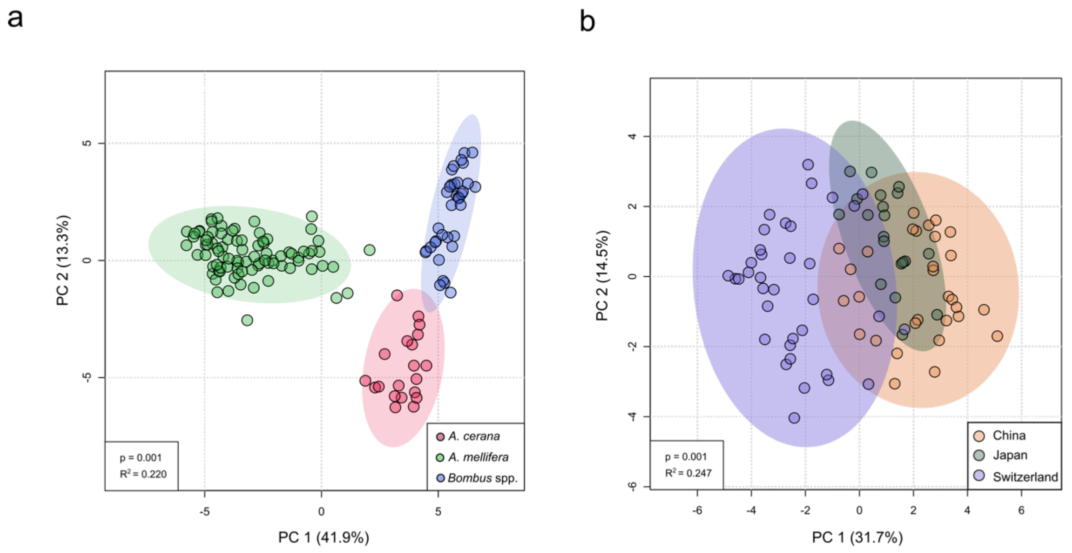

Supplementary Fig. 6. a PCA analysis shows RiPPs distribution in the gut microbiomes of *A. cerana*, *A. mellifera*, and *B. terrestris* individuals. **b** RiPPs of the honeybee gut microbiota are distinguishable between China, Japan, and Switzerland. PCA analysis shows RiPPs distribution in the honeybee gut microbiomes of China, Japan, and Switzerland country individuals. Group differences were tested by one-way permutational multivariate ANOVA (PERMANOVA).

a

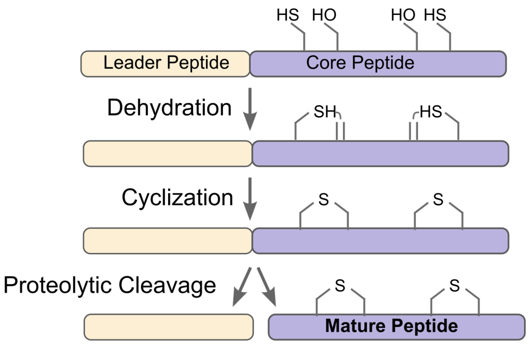

b

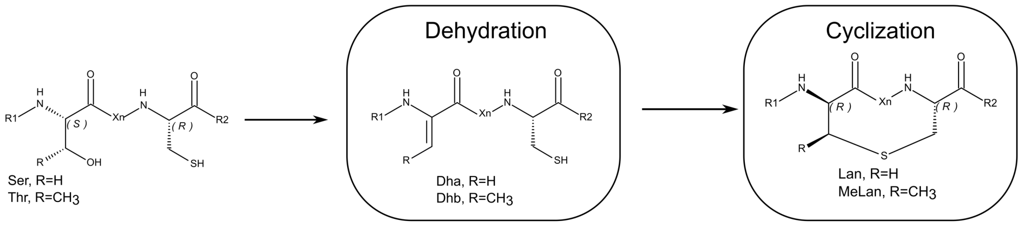

Supplementary Fig. 7. a Processing order of the precursor peptide by the lanthipeptide processing enzyme. **b** Underlying principles of Lanthipeptide II biosynthesis. Dha = Dehydroalanine. Dhb = Dehydrobutyrin

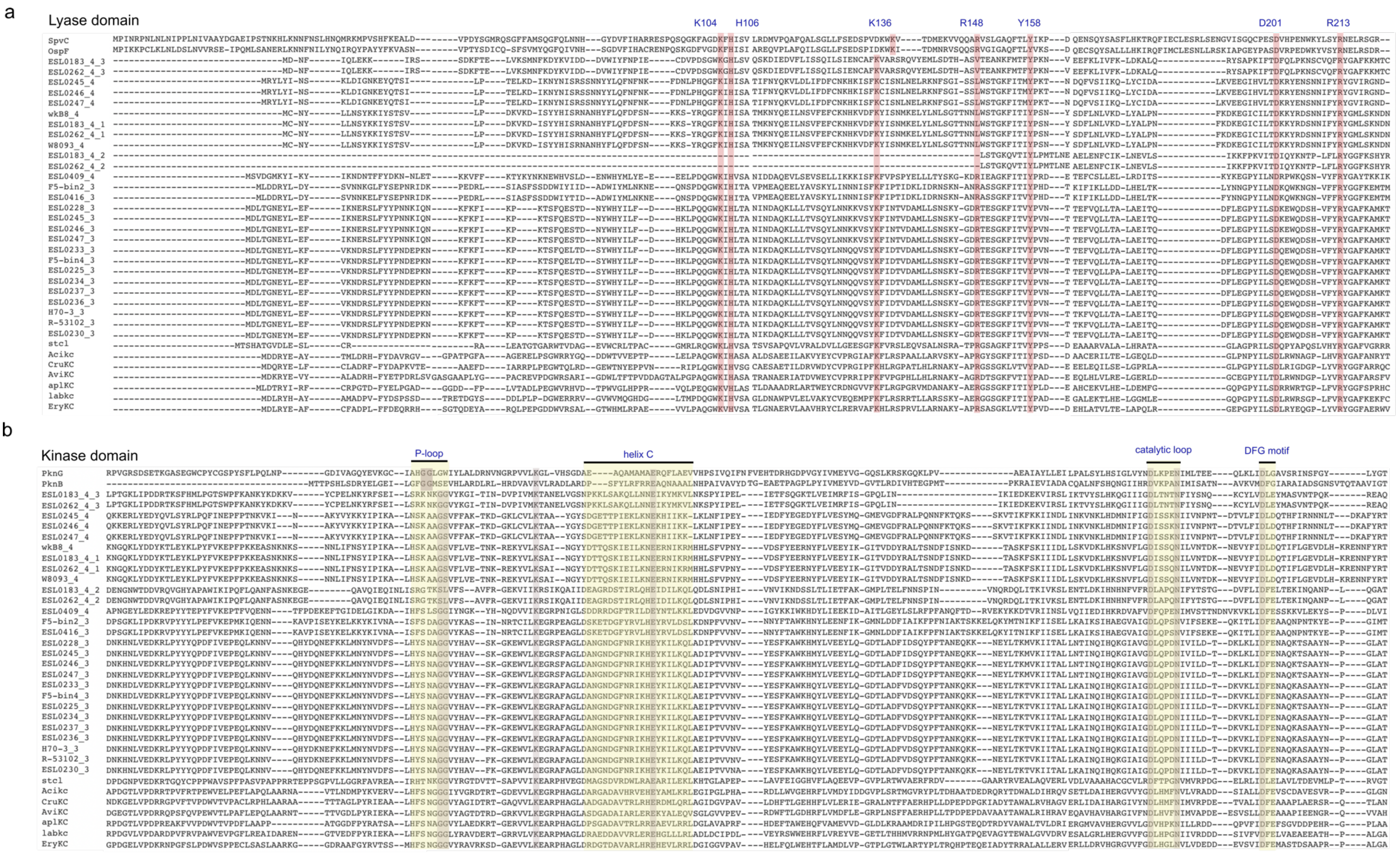
a
Lyase domain
K104
H106
K136
R148
Y158
D201
R213
SpvC
OspF
ESL0183_4_3
ESL0262_4_3
ESL0245_4
ESL0246_4
ESL0247_4
wkB8_4
ESL0183_4_1
ESL0262_4_1
W8093_4
ESL0183_4_2
ESL0262_4_2
ESL0409_4
F5-bin2_3
ESL0416_3
ESL0228_3
ESL0245_3
ESL0246_3
ESL0247_3
ESL0233_3
F5-bin4_3
ESL0225_3
ESL0234_3
ESL0237_3
ESL0236_3
H70-3_3
R-53102_3
ESL0230_3
stcl
AcikC
CruKC
AviKC
aplKC
labkc
EryKC
b
Kinase domain
P-loop
helix C
catalytic loop
DFG motif
PknG
PknB
ESL0183_4_3
ESL0262_4_3
ESL0245_4
ESL0246_4
ESL0247_4
wkB8_4
ESL0183_4_1
ESL0262_4_1
W8093_4
ESL0183_4_2
ESL0262_4_2
ESL0409_4
F5-bin2_3
ESL0416_3
ESL0228_3
ESL0245_3
ESL0246_3
ESL0247_3
ESL0233_3
F5-bin4_3
ESL0225_3
ESL0234_3
ESL0237_3
ESL0236_3
H70-3_3
R-53102_3
ESL0230_3
stcl
AcikC
CruKC
AviKC
aplKC
labkc
EryKC

Supplementary Fig. 8. a Excerpt of an alignment of OspF from *Shigella dysenteriae* and SpvC from *Salmonella paratyphi* with the lyase domains of exemplary Lanthipeptide III and IV synthetases. Above the alignment, the numbering of the catalytic residues of SpvC is shown. **b** The kinase domains of *Lactobacillus* Firm5 shared the conserved residues with exemplary Lanthipeptide III (AciKC, AplKC, LabKC, CruKC, AviKC, EryKC)/IV (StcL) and the mycobacterial protein kinases (PknB, PknG) enzymes[1]. Conserved catalytic residues are shown in grey and structural features important for activity are highlighted.

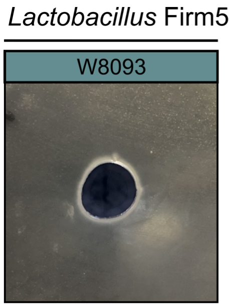

Supplementary Fig. 9. The cell-free supernatant of W8093 does not inhibit the growth of *M. plutonius in vitro*.

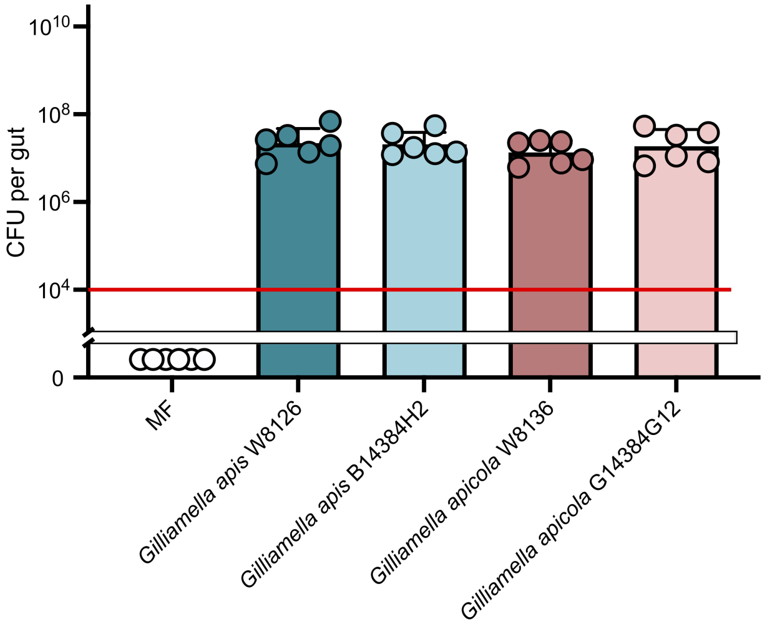

Supplementary Fig. 10. The graph showing the total CFU per gut estimated by bacteria culture for MF honeybees and *G. apis* strains (B14384H2, W8126) and *G. apicola* strains (W8136, G14384G12). Error bars represent mean SD. n=6 biologically independent replicates. Source data are provided as a Source Data file.

Supplementary Table 1. List of peptide sequence. P-Values of the peptide-spectrum matches are computed using MS-Direct Probability Redistribution for estimating Peptide Spectrum Matches based on rare event probability estimation by multilevel splitting [2].

ID	Fragment Seqence	Modified Seqence	Score	P-Value	PeptideMass	SpectrumMass	Charge	FDR
1	KATTSNTISA	KATTSNT-18ISA	6	8.10E-11	974.503	488.253	2	0
2	IFMILTRN	IFMILTRN	6	8.90E-12	1006.56	504.293	2	25
3	MILPI	MILPI	5	2.40E-11	585.356	586.363	1	50
4	YTLF	YT-20LF	5	3.80E-11	522.248	523.244	1	33.33
5	PIYP	PIYP	5	3.90E-11	488.263	489.275	1	25

Supplementary information references

1. Hegemann JD, Sussmuth RD. Matters of class: coming of age of class III and IV lanthipeptides. *RSC Chem Biol* **1**, 110-127 (2020).

2. Mohimani H, Kim S, Pevzner PA. A new approach to evaluating statistical significance of spectral identifications. *J Proteome Res* **12**, 1560-1568 (2013).
