# Supplementary material for: Identification of peptides from honeybee gut symbionts as potential antimicrobial agents against Melissococcus plutonius
Source: Nat Commun. 2023 Nov 24;14:7650. doi: 10.1038/s41467-023-43352-6 (PMC10673953; doi:10.1038/s41467-023-43352-6)
Supplement: Supplementary file 3 — Description of Additional Supplementary Files [file 41467_2023_43352_MOESM3_ESM.docx]

**Description of Additional Supplementary Files**

**File Name: Supplementary Data 1**

**Description:** List of bee gut bacterial genomes.

**File Name: Supplementary Data 2**

**Description:** List of BGCs from the bacterial genomes with known categories.

**File Name: Supplementary Data 3**

**Description:** Biosynthesis gene cluster families (GCFs) and gene cluster clans (GCCs) in the 477 bee gut bacterial genomes based on interactive sequence similarity network analysis using BiG-SCAPE.

**File Name: Supplementary Data 4**

**Description:** The distribution of BGCs across bumble (B. terrestris) and honeybee (A. cerana and A. mellifera) gut metagenomes. The numbers indicate the reads per killobase per million mapped reads to the BGCs.

**File Name: Supplementary Data 5**

**Description:** List of bee gut metagenomes.
