# Supplementary material for: Identification of peptides from honeybee gut symbionts as potential antimicrobial agents against Melissococcus plutonius
Source: Nat Commun. 2023 Nov 24;14:7650. doi: 10.1038/s41467-023-43352-6 (PMC10673953; doi:10.1038/s41467-023-43352-6)
Supplement: Supplementary file 9 — Reporting Summary [file 41467_2023_43352_MOESM9_ESM.pdf]

nature portfolio

Corresponding author(s): Hao Zheng

Last updated by author(s): Oct 31, 2023

Reporting Summary

Nature Portfolio wishes to improve the reproducibility of the work that we publish. This form provides structure for consistency and transparency in reporting. For further information on Nature Portfolio policies, see our Editorial Policies and the Editorial Policy Checklist.

Statistics

For all statistical analyses, confirm that the following items are present in the figure legend, table legend, main text, or Methods section.

n/a	Confirmed	
☐	☒	The exact sample size (n) for each experimental group/condition, given as a discrete number and unit of measurement
☐	☒	A statement on whether measurements were taken from distinct samples or whether the same sample was measured repeatedly
☐	☒	The statistical test(s) used AND whether they are one- or two-sided<br>*Only common tests should be described solely by name; describe more complex techniques in the Methods section.*
☐	☒	A description of all covariates tested
☐	☒	A description of any assumptions or corrections, such as tests of normality and adjustment for multiple comparisons
☐	☒	A full description of the statistical parameters including central tendency (e.g. means) or other basic estimates (e.g. regression coefficient) AND variation (e.g. standard deviation) or associated estimates of uncertainty (e.g. confidence intervals)
☐	☒	For null hypothesis testing, the test statistic (e.g. F, t, r) with confidence intervals, effect sizes, degrees of freedom and P value noted<br>*Give P values as exact values whenever suitable.*
☒	☐	For Bayesian analysis, information on the choice of priors and Markov chain Monte Carlo settings
☒	☐	For hierarchical and complex designs, identification of the appropriate level for tests and full reporting of outcomes
☒	☐	Estimates of effect sizes (e.g. Cohen's d, Pearson's r), indicating how they were calculated

Our web collection on statistics for biologists contains articles on many of the points above.

Software and code

Policy information about availability of computer code

Data collection	For quantitative real-time PCR data collection, QuantStudio Design and Analysis Software (version 1.5.1) are used.
Data analysis	We analyzed genome sequences of bee gut bacterial strains using antiSMASH 5.0. All potential biosynthetic gene clusters (BGCs) were processed through the BiG-SCAPE package, generating interactive BGC sequence similarity networks to distinguish among BGC classes. We assessed the abundance of secondary metabolite gene clusters in honey bee gut microbiomes via the BiG-MAP pipeline. We used MetaMiner to integrate tandem mass spectra and genomic data to identify the RiPP-encoding BGC in Gilliamella.<br>The list of analysis software and all scripts generated for analysis have been deposited on GitHub at: https://github.com/HaoyuLang/Bee_micro_BGC.git

For manuscripts utilizing custom algorithms or software that are central to the research but not yet described in published literature, software must be made available to editors and reviewers. We strongly encourage code deposition in a community repository (e.g. GitHub). See the Nature Portfolio guidelines for submitting code & software for further information.

Data

Policy information about availability of data

All manuscripts must include a data availability statement. This statement should provide the following information, where applicable:

- Accession codes, unique identifiers, or web links for publicly available datasets
- A description of any restrictions on data availability
- For clinical datasets or third party data, please ensure that the statement adheres to our policy

All data generated in this study are provided within the manuscript files. Source Data is provided.

Research involving human participants, their data, or biological material

Policy information about studies with human participants or human data. See also policy information about sex, gender (identity/presentation), and sexual orientation and race, ethnicity and racism.

Reporting on sex and gender	n/a
Reporting on race, ethnicity, or other socially relevant groupings	n/a
Population characteristics	n/a
Recruitment	n/a
Ethics oversight	n/a

Note that full information on the approval of the study protocol must also be provided in the manuscript.

Field-specific reporting

Please select the one below that is the best fit for your research. If you are not sure, read the appropriate sections before making your selection.

☒ Life sciences ☐ Behavioural & social sciences ☐ Ecological, evolutionary & environmental sciences

For a reference copy of the document with all sections, see nature.com/documents/nr-reporting-summary-flat.pdf

Life sciences study design

All studies must disclose on these points even when the disclosure is negative.

Sample size	Sample sizes were chosen based on those used in similar studies to which we expected similar effect sizes in our current experiments (Zheng et al. 2017, PNAS; Zhang et al. 2022, Nat Commun).
Data exclusions	No data were excluded from the analyses.
Replication	For all experiments, at least three biological replicates were used, as noted in the manuscript, to yield reproducible results. All attempts at replication were successful.
Randomization	Bees were randomly assigned to different treatment groups.
Blinding	All analysis were performed blind.

Reporting for specific materials, systems and methods

We require information from authors about some types of materials, experimental systems and methods used in many studies. Here, indicate whether each material, system or method listed is relevant to your study. If you are not sure if a list item applies to your research, read the appropriate section before selecting a response.

Materials & experimental systems

n/a	Involved in the study	
x		Antibodies
x		Eukaryotic cell lines
x		Palaeontology and archaeology
	x	Animals and other organisms
x		Clinical data
x		Dual use research of concern
x		Plants

Methods

n/a	Involved in the study	
x		ChIP-seq
x		Flow cytometry
x		MRI-based neuroimaging

Animals and other research organisms

Policy information about studies involving animals; ARRIVE guidelines recommended for reporting animal research, and Sex and Gender in Research

Laboratory animals	Worker honeybees (Apis mellifera) were used in this study.
Wild animals	The study did not involve wild animals.
Reporting on sex	Our experimental subjects are gender-independent. We performed all experiments with worker honeybees, which are females.
Field-collected samples	We collected honeycombs from the managed honeybee colonies to generate microbiota-free and mono-colonized bees in the lab. All honeycombs of Apis mellifera were collected from the apiary of China Agricultural University, Beijing, China during the Summer and Autumn 2022. Brood frames were collected from a single hives and transfered to the lab.
Ethics oversight	No ethical approval was required in this case.

Note that full information on the approval of the study protocol must also be provided in the manuscript.
